# Performance of different support surfaces during experimental resuscitation (CPR)

**DOI:** 10.1016/j.heliyon.2016.e00074

**Published:** 2016-02-19

**Authors:** Esa Soppi, Ansa Iivanainen, Leila Sikanen, Elina Jouppila-Kupiainen

**Affiliations:** a Eira Hospital, Helsinki, Finland; b Mikkeli University of Applied Sciences, Mikkeli, Finland

**Keywords:** Health profession, Health sciences, Applied sciences

## Abstract

The relationship between the efficacy of resuscitation and the mattresses and backboards used in acute care units, has been studied previously. However, few reports focus on the relative efficacy of resuscitation when using mattresses with different modes of function. This study examines the performance of different support surfaces during experimental cardiopulmonary resuscitation (CPR). The surfaces included a hard surface, a higher specification foam mattress, a dynamic, alternating pressure mattress, and a dynamic, reactive minimum pressure air mattress system. A pressure sensitive mat was placed between the mattresses and each surface and the efficacy of resuscitation measured using differences in compression frequency, compression depth and hands-on time. Our results suggest that the efficacy of resuscitation is dependent on the mode of action of the mattress, while adequate compression frequency and depth do not have a significant effect. In the open system alternating mattress, deflation of the mattress using the CPR function improved the stability of the resuscitation in our study, especially in situations where the height of the air mattress is greater than 20–25 centimeters. Using our experimental system, resuscitation on a closed air system mattress optimally combined stability and effort, while the CPR function converts the air system of the mattress to open, which impairs its functionality during resuscitation. These results indicate that resuscitation is dependent of the mode of action of the mattress and whether the mattress-specific CPR function was used or not. However, the interactions are complex and are dependent on the interaction between the body and the mattress, i.e. its immersion and envelopment properties. Furthermore, this study casts doubt on the necessity of the CPR function in air mattresses.

## 1 Introduction

Patients in intensive care units (ICU) are severely ill, susceptible to pressure ulcers and may need acute cardiopulmonary resuscitation (CPR) [1, 2]. A decade ago, on average 2.7% of European patients in intensive care units required CPR, but the variation across countries and intensive care units was more than ten-fold [2]. Currently, the proportion of patients who need CPR may be similar, although ICU-patients are more ill, since procedures to predict the need for CPR have advanced.

The prevalence of pressure ulcers (PU) in intensive care units has decreased during the last 20 years from about 30% to 10% [1, 3, 4, 5, 6]. This is probably due to a combined result of an increased knowledge of the pathophysiology of pressure ulcer development, advances in therapeutic intensive care methods and preventive measures against PUs, especially the introduction of specialty mattresses.

There is a huge variation in the properties of different mattresses, ranging from static foam to dynamic air mattresses. Different mattresses perform differently in case of CPR [7, 8, 9, 10, 11, 12]. To increase the stability and effectiveness of CPR, a solid plate is often placed between the (foam) mattress and patient [10, 12, 13] Some of the continuously functioning air mattresses are so called open systems which are required by the manufacturer to be deflated when CPR is begun, while others are closed systems and do not require deflation. Air mattresses in acute care units are nevertheless expected to be equipped with a CPR control system for rapid deflation of the air mattress before normal resuscitation procedures are carried out.

Little is known about whether the use of the CPR function in air mattresses really effects on the stability and effectiveness of CPR. We have examined the functionality and performance of different types of mattresses during experimental resuscitation.

## 2 Materials and methods

### 2.1 Mattresses

A triple layer, higher specification polyurethane mattress (HSFM) [14] (EkoUltra^®^, Medimattress Ltd. Helsinki, Finland; dimensions: width, length, height = 80 × 200 × 13 cm) was used as a medium risk antidecubitus mattress.

The dynamic, best (studied) alternating pressure mattress, Nimbus^®^ 3 (ArjoHuntleigh Healthcare, UK; dimensions: width, length, height = 89 × 208.5 × 21.5 cm), was used as an example of a high risk alternating antidecubitus mattress. The air system of the mattress is open, since the pumps of the control unit fill the air cells of the mattress continuously nonstop (24/7). The mattress needs to be deflated for CPR according to manufacturer's instructions.

A dynamic, non-alternating, minimum pressure air mattress system (Carital^®^ Optima) adjusts the three segments of the support surface always optimally to the continuous low pressure with unique functionality and efficacy [3, 14]. The microprocessor guided control unit both measures the conditions within each of the segments of the mattress and adjusts the conditions within the segments (each segment individually to preprogrammed values) of the mattress automatically and individually according to patient's weight, body shape and position. The Carital^®^ Optima with a CPR function (Carital Ltd. Helsinki, Finland; dimensions: Width, length, height = 80 × 200 × 13 cm) was used as an extremely high risk antidecubitus mattress. The CPR function of this mattress deflates the middle section of the mattress. The air system of the mattress is closed, since the pumps of the control unit fill the air cells of the mattress only to the point where the pressure sensors of the control unit indicate that the optimum pressure values in head, middle or foot sections of the mattress is reached. Then the computer directed by the pressure sensors shuts the air pumps and the air valves are closed preventing the air to escape from the mattress. In clinical use, the air pumps of the Carital^®^ function typically only some 30–90 min per day.

All mattresses had their standard covers and the mattresses were used according to the manufacturer's instructions.

### 2.2 Patient simulator manikin

An interactive SimMan^®^ 3G (Leardal Medical, Norway) patient simulator (weight 40 kg) with full patient monitoring and advanced video system package was used to monitor the course of the experimental cardiopulmonary resuscitation according to the manufacturer's instructions. The compression frequency (/min), compression depth (mm, measured with an optoelectronic device within the manikin) and the hands-on time were recorded. The hands-on time is defined as the ratio (x100 = %) between TimeWithFlowDuringPerfusionStop and TimeWithPerfusionStop, where TimeWithPerfusionStop is the time the heart is not pumping blood (no effective heart rhythm, i.e. the entire resuscitation cycle). TimeWithFlowDuringPerfusionStop is the time when the compressions, i.e. heart is pumping, are recorded during perfusion stop (during the resuscitation cycle). Compressions are recorded when the compression depth changes by 5 mm or more during the previous 2 seconds at a continuous pace of more than 0.25 compressions/sec (hands-on time definition: Personal communication from Leardal).

### 2.3 Interface pressure and contact area measurements

The FSA Pressure Mapping Systems (a thin mat, about 1 mm thickness with width x length= 90 × 200 cm equipped with 64 × 32 = 2048 pressure sensors) (Vista Medical, Canada, program version FSA 4.0) was used to measure the mean interface pressure and contact area between the mattresses and the SimMan^®^ manikin.

The FSA System was throughout all experiments placed between the surface and the SimMan^®^ manikin.

The FSA Systems was calibrated before the study and used according to the manufacturer's instructions. Continuous recording was started 30 seconds before the command “Start resuscitation” with the manikin lying on the hard floor on EkoUltra and Carital^®^ mattresses, respectively. The recordings were collected at time points 5, 15 and 25 seconds before the command “Start resuscitation”. The recording time on Nimbus^®^ was 60 seconds before the command. The recordings were collected at time points 5, 15, 25, 35, 45 and 55 seconds before the command “Start resuscitation”. For analysis of the results the command “Exclude minimum” from the analysis program was chosen; this excludes the non-contact sensors between the FSA mat and the manikin and eliminates meaningless background data from the analysis [15]. Before the command “Start resuscitation” the mean interface pressure, variance and contact area values were recorded. Variance was defined a measure of the variability, or dispersion, of individual sensor values around the average pressure value and it was recorded as the arithmetic mean of the squared deviations from the mean. After the “Start resuscitation” command, the lowest and highest interface values around the defined time points together with the corresponding variance and contact area values were retrieved.

### 2.4 Procedures

Resuscitation was performed by nursing students (4 females, 2 males: Age range 24–36 yrs; height range 161–176 cm; weight range 59–75 kg). They had got basic training in CPR and all had had practical experience on CPR in their work. They were fully informed about the test procedures in advance. This included practice in doing experimental CPR with the SimMan^®^ manikin; they were to reach a resuscitation frequency of 100–120/min and a chest compression depth of at least 5 centimeters [13]. During the actual testing no advice or feedback on either parameters were given to the resuscitators. They were required to rest at least 20 min between each CPR session.

Each CPR session lasted for only 2 min to exclude any performer fatigue. The frequency, chest compression depth and hands-on time were recorded and retrieved at the time points 5, 30, 60 90 and 120 seconds during resuscitation. At the same time points, the minimum and maximum mean interface pressure values (mmHg) and the minimum and maximum contact surface values (cm^2^) between the manikin and the mattress were recorded.

CPR was performed on the hard, flat floor of the test laboratory on which all mattresses were placed. The resuscitators were on their knees during CPR on a soft surface with a thickness at the level of the top of each mattress. The procedure was chosen to exclude the possible confounding effect of the bed.

CPR was carried out on the manikin as follows: a) the higher specification foam mattress (EkoUltra^®^), b) the Carital^®^ after stabilization, c) the Carital^®^ after stabilization and CPR valve opened at the command “Start resuscitation”, d) Nimbus^®^ with a comfort soft, in static mode, e) Nimbus^®^ with a comfort soft, in static mode with CPR control valve opened at the command “Start resuscitation”, f) Nimbus^®^ with a comfort soft, in alternating mode, g) Nimbus^®^ 3 with a comfort soft, in alternating mode with CPR control valve opened at the time of the command “Start resuscitation”, h) Nimbus^®^ with a comfort hard, static mode as an internal control, i) Nimbus^®^ with a comfort hard, alternating mode as an internal control. These modes are defined in the user manual of the mattress.

The dynamic mattresses were allowed to recover and stabilize after each CPR session to the point where their indicator lights showed that they were ready for use. Thereafter, the basal levels of the mean interface pressures and contact areas were recorded for 30 seconds in case of hard surface, foam and Carital^®^ mattress and for one minute when the dynamic Nimbus^®^ mattress were used.

After each CPR session, the resuscitators were asked to mark the stability of the resuscitation procedure on visual analog scale (VAS, 0–100 mm), where maximum stability (100 mm on the VAS scale) was attained when the manikin was placed on the FSA Pressure Mapping Systems which lay on a hard, flat floor.

After each CPR session the resuscitators were asked to quantify in words how strenuous they considered the CPR effort to have been.

### 2.5 Ethical issues

Ethical committee review was not required. The nursing students gave their verbal consent to participate.

### 2.6 Statistical methods

Study variables were analyzed using analysis of variance (ANOVA) models. Repeated measurement ANOVA models were used for most of the variables in order to explore the time effect in addition to mattress type. 2-sided p-value of 0.05 was considered as statistically significant in the analyses although no actual hypothesis were set for the study. Anyway, as the study was rather small sized (6 nursing students performing the resuscitation), this usual significance level was considered as an adequate signal of an effect, taking into account also the clinical significance of observed differences. To compare exclusively the performance of mattresses the hard, flat floor was chosen to eliminate the probable confounding effect of the bed. The focus in the comparisons was in the differences between Carital^®^ Optima and the other products. Analyses were conducted using proc mixed of SAS(R) software version 9.1.3.

## 3 Results

The achieved compression frequency was significantly higher on hard floor than on Carital^®^ Optima. Compression frequency was also markedly lower on Nimbus (except comfort control hard, alternating) than on Carital^®^ (Table 1).

Compression depth of Carital^®^ was significantly greater than on Carital Optima with CPR function in use (*P* = 0.0056). The corresponding values Nimbus^®^ (hard comfort, alternating mode) were significantly higher than on Carital^®^ (*P* = 0.0029) (Table 1). The difference with other modes of Nimbus^®^ did not differ from that of Carital^®^ Optima.

Hands on time, describing the efficiency of resuscitation, on Carital^®^ was not different from hard floor, HSFM, Carital^®^ with CPR function in use or Nimbus^®^ (comfort control, static mode) (Table 1). Hands on time on Carital^®^ was significantly higher than on any other Nimbus modes (Table 1).

The stability of the experimental resuscitation on different mattresses and set-ups was compared to the situation when the manikin was placed on the hard, flat floor. The higher specification foam mattress performed closest to the hard surface, followed by the Carital^®^ Optima with a closed air compartment system and Nimbus^®^ comfort control soft, in alternating mode with CPR function in use (Table 2). The stability of Carital^®^ vs. Carital^®^ with CPR control function in use was not statistically significant (*p* = 0.539). The stability of the Nimbus^®^ 3 was considerably less although deflation by the CPR control did increase the stability of the resuscitation procedure (Table 2).

The test persons experienced progressively harder strain of the CPR when the resuscitation was performed on any of the Nimbus^®^ mattress modes.

At baseline, before the start of CPR, the mean interface pressure was highest and the contact area lowest on the hard floor (*p* < 0.001, Table 3). The next highest interface pressure values were encountered on the Nimbus^®^ mattress with the comfort control in the static hard mode (*p* < 0.001 compared to Carital Optima, Table 3). The contact areas on HSFM and Nimbus (comfort control soft and hard, both in alternating mode) compared to Carital^®^ were significantly lower, *p* = 0.0231, 0.0752 and *p* = 0.0148, respectively. Setting the Nimbus^®^ to alternating mode tended to decrease the contact area as compared to the static mode (Table 3).

After the start of CPR, the mean maximum interface pressure values were the highest and the contact area the lowest between the manikin and FSA mat on the hard floor (Table 4), followed by the Nimbus^®^ with comfort control soft either in static or alternating mode, CPR valves open at start of CPR (*p* < 0.0001 in all cases, Table 4). The next highest interface pressures were recorded on the Nimbus^®^ 3 with the comfort control in soft setting in either static or alternating mode. The contact areas with the EkoUltra^®^ foam mattress were somewhat lower than with the Nimbus^®^ mattress in the above situations, but the mean interface pressure was markedly lower. The interface pressure and contact area values with the Carital^®^ resembled those of the Nimbus^®^ with the comfort control in soft setting in either static or alternating mode (Table 4).

## 4 Discussion

Even if differences in compression frequency or compression depth between different surfaces were observed, the efficiency of resuscitation in this experimental situation seems adequate, irrespective of the mattress or whether the CPR function of the mattress was in use or not. The results were of the same magnitude as in previous studies [9, 16]. However, there were differences in the hands-on time indicating that the frequency and depth values in all situations are not of similar significance: the different settings of the Nimbus^®^ mattress differ from each other with respect to the resuscitation efficiency [8, 9]. However, the results between hard floor, HSFM and Carital were comparable.

The stability of the resuscitation procedure on Nimbus^®^ was inferior to that of the foam mattress or the Carital^®^ minimum pressure mattress, which is probably due to the thickness of Nimbus^®^ 3 (21.5 cm) compared with the higher specification foam and Carital^®^ mattresses (both 13 cm). Use of the CPR function improved the stability of the Nimbus^®^ mattress, probably due to deflation of the mattress which made it thinner and made the setting resemble the other two mattresses. The resuscitators also felt that resuscitation on the Nimbus mattress required more effort than the other mattresses which is also attributable to the thickness difference between the Nimbus^®^ and the other mattresses. The ensuing fatigue may be due to fact that the resuscitators needed to move their hands downwards with more force than with the other mattresses to achieve sufficient chest compression. The strain may further increase by the air escaping from the open system of the Nimbus^®^ during resuscitation. This hypothesis supported by the finding that use of the CPR function with the Carital^®^ mattress, which makes the mattress air system open, tended to deteriorate the stability of the mattress.

To gain insight into the functionality of the mattresses, the mean pressure values and contact area of the interface were measured using the FSA mat. The interface pressure was highest and the contact area lowest on hard surface, as expected. With the comfort control set at “hard”, the Nimbus^®^ produced higher interface pressure values than the other mattresses. Contact area with Carital Optima was highest; the difference was not significant when compare to static modes of the Nimbus^®^ mattress. However, when its alternating modes were introduced, the contact areas decreased as expected as only a smaller part of the mattress is in the contact with the manikin/patient (Table 3). This most probably reflects the ability of the mattresses to control the immersion and envelopment of the manikin (Table 3) [11, 14, 17, 18]. The difference is, however, smaller than expected, which may be due to at least two reasons. Firstly, the manikin weighs only about 40 kilograms which is much less than the weight of the ordinary adult patient. Secondly, the FSA mat is not very flexible and this may prevent it from adapting itself closely to the contours of the mattress. In previous experiments we have noticed this to be in case, and we suspect that the current FSA mattress averages out both low and high contact areas although it shows the trends correctly. Still, the differences between various mattresses regarding contact areas also reflect differences in their pressure redistribution properties. Further, the pressure redistribution is a function of the immersion and envelopment properties of various mattresses [8, 16, 17, 19]. The combined effect of immersion and envelopment is apparently close to the ideal values in the Carital^®^ [14, 18]. In real life the differences are expected to be even larger since human body is heavier and complete as compare to the manikin.

Nimbus^®^ with the CPR function in use during resuscitation produced the highest interface pressure values and the lowest contact areas, i.e., this mattress required more effort from the resuscitator for the same compression depth than the other mattresses. This is in line with the resuscitators' experience, according to which resuscitation on the Nimbus^®^ required more effort and felt strenuous. Hands on time reflecting the efficacy of resuscitation were lower on Nimbus^®^ 3 and decreased further when CPR function was taken into use (Table 1). This is line with recent findings by Sainio et al. (2014) [20], who also noticed that use of CPR function in another alternating mattress deteriorated the efficacy of resuscitation.

The results in the alternating mode of the Nimbus^®^ were next in order as concerns interface pressure values, while the corresponding minimum contact areas were close to that of the foam mattress. As all cells of the Nimbus^®^ mattress came into contact with the FSA mat, the contact areas became maximal and approached the values observed with Carital^®^. The Carital^®^ mattress produced low interface pressure values concomitantly with high contact areas and this is in line with the experience of the resuscitators, who stated that lower energy was needed to produce a compression depth similar to various Nimbus^®^ mattresses settings.

Taken together the results indicate that an adequate compression frequency and depth are achieved without the use of the CPR function of the air mattresses [9]. Foam mattresses with higher specifications perform well and do not need a supporting hard, flat surface inserted between the mattress and patient. Still, such a hard plate may be needed when conventional hospital foam mattresses are used, but such mattresses were outside the scope of this study. In the open systems, represented here by the Nimbus^®^, deflation of the mattress with the CPR function seems, however, to improve the stability of resuscitation, especially in the situations where the height of the air mattress exceeds 20–25 centimeters. Resuscitation with a closed air system, e.g., the Carital^®^ mattress, seems to produce an optimal combination with respect to stability and effort. Use of the CPR function in this type of mattress makes the mattress air function an open system and this only impairs performance during resuscitation.

Limitations of the study include relatively low number of test person and lack of randomization, which however was not carried for practical reasons i.e. 20 min between each CPR session could have not been guaranteed. The test persons were not given feedback during the resuscitation sessions which might have influence on the results of compression frequency and depth i.e. the quality of CPR of the rescuer. A feedback device to measure the total compression deliver by the rescuer could have been introduced. However, these limitations most probably have not a major effect of the results and conclusions. Still it needs to be remembered that rarely resuscitation is carried out on hard floor but on a bed. Furthermore, the rescuer is not on their knees; it is standing.

## Declarations

### Author contribution statement

Esa Soppi, Ansa Livanainen: Conceived and designed the experiments; Performed the experiments; Analyzed and interpreted the data; Contributed reagents, materials, analysis tools or data; Wrote the paper.

Leila Sikanen, Elina Jouppila-Kupiainen: Conceived and designed the experiments; Performed the experiments; Analyzed and interpreted the data; Contributed reagents, materials, analysis tools or data.

### Funding statement

The authors received no funding from an external source. Carital Group funded the statistical analysis performed by an independent company, 4Pharma Ltd.

### Conflict of interest statement

The authors declare the following conflict of interests: Esa Soppi is the Chairman of the Board of Carital Group.

### Additional information

No additional information is available for this paper.

## Figures and Tables

**Table 1:** Effect of mattress type on the compression frequency, compression depth and hands-on time.

		Flat hard floor	A higher specification foam mattress (HSFM)	Carital^®^ Optima	Carital^®^ Optima with CPR control function in use i.e. CPR valve opened	Nimbus^®^ 3 comfort control soft, static mode	Nimbus^®^ 3 comfort control soft, static mode with CPR control function in use	Nimbus^®^ 3 comfort control soft, alternating mode	Nimbus^®^ 3 comfort control soft, alternating mode with CPR control function in use	Nimbus^®^ 3 comfort control hard, static mode	Nimbus^®^ 3 comfort control hard, alternating mode
		1	2	3	4	5	6	7	8	9	10
Compression frequency /min (SD)	A	121 (11)	110 (8)	109 (9)	108 (5)	105 (8)	105 (12)	106 (10)	105 (6)	106 (8)	109 (8)
Compression depth, mm (SD)	B	46 (5)	43 (8)	46 (7)	42 (11)	46 (6)	48 (9)	48 (7)	47 (6)	47 (6)	49 (7)
Hands-on time % through 5-120s (SD)	C	83 (24)	84 (24)	83 (24)	85 (24)	78 (23)	62 (28)	62 (18)	51 (23)	51 (12)	56 (10)
N*		25	25	30	25	30	28	25	27	25	24
p-value (CI 95%)	A	<0.0001 (8.5,14.5)	0.9893 (−3.0,3.0)	– –	0.1985 (−4.9,1.1)	0.0088 (−6.8,−1.0)	0.0054 (−7.1,−1.3)	0.0652 (−5.8,0.2)	0.0007 (−8.2,−2.4)	0.0321 (−6.3,−0.3)	0.9875 (−3.1,3.0)
p-value (CI 95%)	B	0.3656 (−3.4,1.3)	0.1700 (−3.9,0.7)	– –	0.0056 (−5.7,−1.0)	0.9758 (−2.1,2.2)	0.0572 (−0.1,−4.4)	0.1078 (−0.4,4.2)	0.1702 (0.7,−3.8)	0.1312 (−0.6,4.1)	0.0029 (1.3,6.1,)
p-value (CI 95%)	C	0.9292 (−7.4,8.1)	0.6919 (−6.2,9.3)	– –	0.5675 (−5.5,10.0)	0.1981 (−12.1,2.6)	<.0001 (−27.6,−12.6)	<.0001 (−25.9,−10.4)	<.0001 (−38.3,−23.1)	<.0001 (−37.3,−22.2)	<.0001 (−32.5,−16.7)

The comparisons are presented in the statistical table (above).

When CPR control valves of both Carital^®^ and Nimbus^®^ mattresses were opened, the air continued to flow out throughout the whole 120 second resuscitation period.

Compression frequency was significantly higher on hard floor than on Carital^®^ Optima. The estimated difference was 11.5 (CI 95% 8.5,14.5; *p* < 0.0001), repeated measurement analysis of variance (point A1).

Compression frequency was close to or significantly lower on Nimbus^®^ than on Carital^®^ Optima except in one mode (point A10).

Mean compression depth decreased somewhat by course of resuscitation (the time effect, *p* = 0.033).

Compression depth of Carital^®^ Optima was significantly greater than on Carital^®^ Optima with CPR function in use. The estimated difference was 3.4 mm (point B4).

The compression depth on Nimbus 3 (comfort hard, alternating mode) were significantly higher (3.7 mm) than on Carital^®^ Optima (point B10).

Mean hands on time increased significantly (mean 10.8 % units) by the course of resuscitation (the time effect, *P* < 0.001).

Hands on time, describing the efficiency of resuscitation, on Carital^®^ Optima was not different from hard floor, HSFM, Carital^®^ Optima with CPR function in use or Nimbus^®^ 3 with comfort control soft, static mode. In all other combinations hands on time on Carital^®^ Optima was significantly higher than on all other Nimbus^®^ modes (*P* < 0.0001)(points C6-10).

* N is based on the repeated measurements as described in the materials and methods section.

**Table 2:** Stability of different mattresses during experimental resuscitation, visual analog scale (VAS)*.

	Flat hard floor	A higher specification foam mattress (HSFM)	Carital^®^ Optima	Carital^®^ Optima with CPR function in use	Nimbus^®^ 3 comfort control soft, in static mode	Nimbus^®^ 3 comfort control soft, in static mode with CPR function in use	Nimbus^®^ 3 comfort control soft, in alternating mode	Nimbus^®^ 3 comfort control soft, in alternating mode with CPR function in use	Nimbus^®^ 3 comfort control hard, in static mode	Nimbus^®^ 3 comfort control hard, in alternating mode
VAS, mm (SD)	100 (−)	82.7 (11.2)	71.2 (20.5)	65.5 (21.8)	32.7 (25.1)	50.8a (27.9)	40.2 (26.4)	71.3b (22.1)	43.8 (24.8)	44.3 (9.7)
N	6	6	6	6	6	6	6	6	6	6

The stability was the lowest on Nimbus comfort control soft, in static mode compared to Carital^®^ Optima. The estimated difference was 38.5 mm (CI 95% 11.8,65.2; *p* = 0.006), repeated measurement analysis of variance. When CPR function was in use on Nimbus^®^ the differences to Carital^®^ Optima were not significant; a) *p* = 0.158 and b) *p* = 0.989.

* VAS where 100 mm = highest stability which was predefined for the hard flat floor and 0 mm represented the lowest stability.

**Table 3:** Mean interface pressure and contact area at baseline before the start of resuscitation.

		Flat hard floor	A higher specification foam mattress (HSFM)	Carital^®^ Optima	Nimbus^®^ 3 comfort control soft in static mode	Nimbus^®^ 3 comfort control soft in alternating mode	Nimbus^®^ 3 comfort control hard in static mode	Nimbus^®^ 3 comfort control hard in alternating mode
Group		1	2	3	4	5	6	7
Mean interface pressure, mmHg (SD)	A	75.1 (2.2)	45.1 (4.3)	44.7 (3.7)	47.6 (6.8)	43.7 (4.0)	59.2 (7.3)	51.5 (7.0)
Contact area, cm^2^ (SD)	B	678 (62)	1109 (449)	1286 (350)	1251 (251)	1149 (203)	1237 (188)	1094 (205)
N*		18	18	18	18	18	18	18
p-value (CI 95%)	A	<0.0001 (−33.6,−27.3)	0.7641 (−3.6,2.7)	– –	0.0651 (−6.1,0.2)	0.5492 (−2.2,4.1)	<0.0001 (−17.6,−11.4)	0.0001 (−10.0,−3.7)
p-value (CI 95%)	B	<0.0001 (457,759	0.0231 (26,329)	– –	0.6363 (−116,187)	0.0752 (−15,288)	0.5110 (−102,201)	0.0148 (40,343)

The comparisons are presented in the statistical table (above).

The mean interface pressure values were the highest on hard flat floor and Nimbus^®^ 3 comfort control hard in static mode. The estimated difference to Carital^®^ Optima was 30.5 and 14.5 mmHg (points A1 and A6) respectively.

The contact area was correspondingly lowest on hard flat floor. The mean difference to Carital^®^ Optima was 608 cm^2^ (Point B1).

* N is based on the repeated measurements as described in the materials and methods section.

**Table 4:** Maximum mean interface pressure, and the corresponding contact area values during experimental resuscitation.

		Flat hard floor	A higher specification foam mattress (HSFM)	Carital^®^ Optima	Carital^®^ Optima with CPR function in use	Nimbus^®^ 3 comfort control soft, in static mode	Nimbus^®^ 3 comfort control soft, in static mode with CPR function in use	Nimbus^®^ 3 comfort control soft, in alternating mode	Nimbus^®^ 3 comfort control soft, in alternating mode with CPR function in use	Nimbus^®^ 3 comfort control hard, in static mode	Nimbus^®^ 3 comfort control hard, in alternating mode
Group		1	2	3	4	5	6	7	8	9	10
Mean maximum interface pressure, mmHg (SD)	A	77.2 (2.0)	63.1 (4.1)	65.3 (2.2)	63.1 (1.8)	65.5 (5.3)	73.1 (4.0)	64.7 (4.3)	71.1 (5.6)	71.4 (5.0)	65.4 (4.3)
Corresponding max contact area, cm^2^ (SD)	B	952 (49)	1885 (110)	2241 (140)	2157 (201)	2048 (146)	1531 (134)	2205 (219)	1723 (323)	2049 (170)	2094 (251)
N*		24	24	24	24	24	24	24	24	24	24
p-value (CI 95%)	A	<0.0001 (9.7,14.0)	0.0425 (−4.4,−0.1)	– –	0.0480 (−4.3,0.0)	0.8821 (−2.0,2.3)	<0.0001 (5.7,10.0)	0.5539 (−2.8,1.5)	<0.0001 (3.7,7.9)	<0.0001 (4.0,8.2)	0.9533 (−2.1,2.2)
p-value (CI 95%)	B	<0.0001 (−1397,−1181)	<.0001 (−464,− 248)	– –	0.1241 (−192,24)	0.0008 (−301,−85)	<0.0001 (−818,−602)	0.5000 (−145,72)	<0.0001 (−626,−410)	0.0008 (−301,−84)	0.0088 (−255,-39)

The comparisons are presented in the statistical table (above).

Mean maximum interface pressure increased (mean 2.7 mmHg) by course of resuscitation (the time effect, *p* = 0.008), repeated measurement analysis of variance.

Mean maximum contact area increased slightly (mean 88 cm^2^) by course of resuscitation but the difference was not significant (the time effect, *P* = 0.087), repeated measurement analysis of variance.

* N is based on the repeated measurements as described in the materials and methods section.
